# Case of Fracture of the Skull

**Published:** 1872-02

**Authors:** Charles W. Saunders

**Affiliations:** Belfast, N. Y.


ART. V.—*Case of Fracture of the Skull.* By CHARLES W. SAUNDERS, M. D. Belfast, N. Y.

At midnight on the 27th of August last, I was called into the town of Lynden, twenty miles distant, to the bedside of an only son, five years of age, of a clergyman of this place, who just at evening of that day was kicked by a horse squarely in the forehead, fracturing the frontal bone horizontally just over the orbits entirely across, and remaining attached in this extent, broke away above in an eliptical form one and one-half inches above the lower fracture, reaching back into the brain substance, this upper margin being depressed one and one-half inches, rupturing the brain largely, about one ounce of which was detached.

I arrived at three o'clock in the morning with my student, Mr. Hopper, meeting Dr. R. Y. Charles, of Rushford, who was faithfully administering to the child's relief. With their assistance, the little fellow being chloroformed, I proceeded to elevate the depressed plate, removing any speculæ and broken down brain matter, so that the edges of bone were nicely in apposition when we dressed up the wound. Anæsthetic effect passing off the little fellow recovered his sense perfectly, which I should have said he had when we arrived, being perfectly conscious from soon after the receipt of the injury.

Dr. Charles took charge of the case; my brother, Dr. J. H. Saunders, and I visiting the patient several times with him thereafter. The little fellow continued to maintain his senses and do reasonably well for about four weeks, after which time he gradually became more irritable, emaciated, and died of gradual exhaustion about eight weeks from the receipt of the injury, having his his senses until near the last, no paralysis being developed. Death seemed to be the passive result of the withdrawal of so much vital force consequent upon the loss of so much brain substance.

